# Neuron-binding antibody responses are associated with Black ethnicity in multiple sclerosis during natalizumab treatment

**DOI:** 10.1093/braincomms/fcad218

**Published:** 2023-08-14

**Authors:** Kiel M Telesford, Chad Smith, Marcel Mettlen, Melissa B Davis, Lindsay Cowell, Rick Kittles, Timothy Vartanian, Nancy Monson

**Affiliations:** Weill Cornell Medicine, Brain and Mind Research Institute, New York, NY 10065, USA; University of Texas Southwestern Medical Center, O’Donnell Brain Institute, Dallas, TX 75390, USA; University of Texas Southwestern Medical Center, Department of Cell Biology, Dallas, TX 75390, USA; Morehouse School of Medicine, Department of Community Health and Preventative Medicine, Atlanta, GA 30310, USA; University of Texas Southwestern Medical Center, Peter O-Donnell Jr. School of Public Health, Dallas, TX 75390, USA; Morehouse School of Medicine, Institute of Genomic Medicine, Atlanta, GA 30310, USA; Weill Cornell Medicine, Brain and Mind Research Institute, New York, NY 10065, USA; University of Texas Southwestern Medical Center, O’Donnell Brain Institute, Dallas, TX 75390, USA

**Keywords:** multiple sclerosis, B cell, autoreactive antibodies, natalizumab, ethnicity

## Abstract

Multiple sclerosis is an inflammatory degenerative condition of the central nervous system that may result in debilitating disability. Several studies over the past twenty years suggest that multiple sclerosis manifests with a rapid, more disabling disease course among individuals identifying with Black or Latin American ethnicity relative to those of White ethnicity. However, very little is known about immunologic underpinnings that may contribute to this ethnicity-associated discordant clinical severity. Given the importance of B cells to multiple sclerosis pathophysiology, and prior work showing increased antibody levels in the cerebrospinal fluid of Black-identifying, compared to White-identifying multiple sclerosis patients, we conducted a cohort study to determine B cell subset dynamics according to both self-reported ethnicity and genetic ancestry over time. Further, we determined relationships between ethnicity, ancestry, and neuron-binding IgG levels. We found significant associations between Black ethnicity and elevated frequencies of class-switched B cell subsets, including memory B cells; double negative two B cells; and antibody-secreting cells. The frequencies of these subsets positively correlated with West African genetic ancestry. We also observed significant associations between Black ethnicity and increased IgG binding to neurons. Our data suggests significantly heightened T cell-dependent B cell responses exhibiting increased titres of neuron-binding antibodies among individuals with multiple sclerosis identifying with the Black African diaspora. Factors driving this immunobiology may promote the greater demyelination, central nervous system atrophy and disability more often experienced by Black-, and Latin American-identifying individuals with multiple sclerosis.

## Teaser

Heightened multiple sclerosis severity exhibited by Black-identifying patients is understudied. Telesford *et al*. report that self-reported Black ethnicity is associated with increased circulating B cell levels, and antibodies that bind to central nervous system neurons. Increased B cell activity may contribute to heightened multiple sclerosis severity for Black-identifying patients.

## Graphical Abstract

**Graphical abstract:**
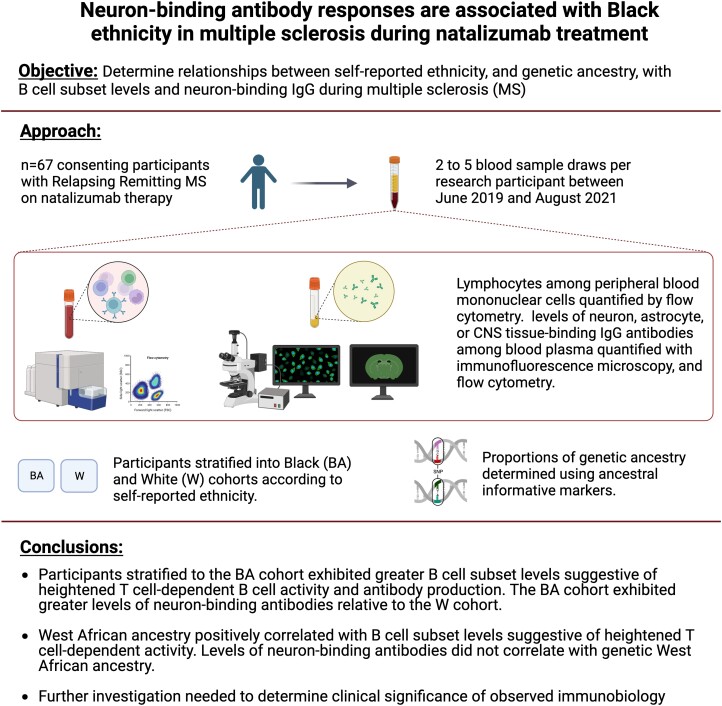

## Introduction

Multiple sclerosis (MS) is an autoimmune, neurodegenerative condition of the CNS often manifesting clinically as sensory, motor or cognitive dysfunction and disability.^1^ Ethnicity is an important influencing factor in MS prognosis, with people that self-identify as ‘Black/African American’, or ‘Hispanic/Latin American’ more likely to experience a poorer prognosis.^2‐6^ Social-determinants of health undoubtedly impact disparate severity among race and ethnicity groups,^7‐10^ however, very little is known about possible biological factors that foster differential outcomes.

Though biological drivers of MS aetiology and pathogenesis are complex, relapsing-remitting MS features an inflammatory infiltrate that traffics into the CNS from the periphery. Circulating B and T lymphocytes in particular are major contributors to MS immunopathogenesis, as evidenced by the efficacy of therapeutic interventions that either specifically deplete circulating B-cells and T-cells (Ocrelizumab, Alemtuzumab),^11,12^ or that limit the trafficking of these cells into the brain [Natalizumab (NAT)].^13^ This central role for lymphocytes in MS pathophysiology highlights related immunobiology as a plausible biological contributor to ethnicity-associated MS severity. Yet, scant data exists on ethnicity-associated differential immunologic trends in MS and potential relationships to heightened severity.

Humoral responses represent a particularly promising avenue of investigation, as mounting evidence demonstrates immunoglobulins to be a correlate of inflammatory demyelination,^14‐16^ CNS degeneration^17,18^ and disability^19^ in MS. Further, three observational retrospective studies show elevated intrathecal immunoglobulin levels for individuals identifying with Black/African American ethnicity compared to those identifying as White,^17,20,21^ possibly linking ethnicity-associated immunoglobulin trends with CNS atrophy.^17^ Our own recent cross-sectional report directly demonstrates that individuals with MS identifying as Black/African American or Latin American, exhibit significantly greater frequencies of antibody-secreting cells (ASCs) compared to White-identifying individuals.^22^ Any relationship between ethnicity and the prevalence of autoreactive antibodies in MS remains unreported in the literature.

In addition to the paucity of published data available for ethnicity-associated immune responses in MS, self-reported identity is often the sole method implemented to distinguish cohorts. While helpful in characterizing larger clinical disparity, and some immunologic trends, socially constructed racial categories are nebulous and broadly defined. This may be particularly limiting when measuring subtle translationally relevant immunological features among populations that are more likely to identify with multiple race/ethnicity categories. Thus, in the present study, we determined relationships between self-identified Black ethnicity, and West African (WAA) genetic ancestry, with several T cell and B cell mediators that are central to driving humoral immunity, demyelination, degeneration, and disability associated with MS. In addition, we provide the first evidence of ethnicity-associated discordant neuronal-reactive antibody levels in MS.

## Materials and methods

### Study participant consent and criteria

We recruited eligible participants from the Weill Cornell Medicine MS center according to Institutional Review Board-approved protocol #1508016490R003. Study participants represent a convenience sample comprising individuals with clinically definite MS according to the 2010 McDonald criteria.^23^ Consenting participants provided informed written consent at the Weill Cornell Medicine MS Center before study inclusion. Eligible participants were clinically stable, and on NAT therapy, having received at least two infusions at the time of the initial blood sample draw. We focused our investigation on NAT-treated participants for several reasons. Our approach enabled the study of intact ethnicity-associated humoral response dynamics^24^ during MS^22^ over time. This design also facilitated the longitudinal examination of lymphocytes that would have otherwise trafficked into the CNS. Before inclusion, participants had not received lymphocyte depletion therapies such as alemtuzumab or ocrelizumab. Participants had largely been treated with other disease modifying therapies before undergoing a washout phase and initiating treatment with NAT.

### Ethnicity cohort stratification

Study participants were asked to self-report ethnicity through a survey instrument. We stratified participants according to self-reported ethnicity into either Black African (BA), or White (W), study cohorts according to the multiple studies demonstrating significant associations between heightened clinical and radiological disease severity with self-identified Black/African American ethnicity, when compared to self-identified White ethnicity. Individuals identifying with more than one categorical ethnicity, (for instance, selecting Latin American/Hispanic ethnicity (LA) in addition to selecting either BA or White ethnicity) were stratified to either BA or W cohorts depending on their selection of Black or White categories. Those identifying solely with LA/Hispanic ethnicity without also indicating Black or White categories survey categories (*n = 5*) were excluded from the categorical analysis.

### Peripheral blood sample processing

Up to five sample draws (SD) were performed for each participant over the course of 26 months between June 2019 and August 2021. We isolated peripheral blood mononuclear cells through density-gradient Ficoll centrifugation. Buffy coats were harvested within hours of peripheral blood collection and were re-suspended in volumes of standard staining buffer equal to the original whole blood. Plasma was separated from peripheral blood aliquots for some blood draws through centrifugation, and stored at −80 degrees for later analysis.

### Genotyping and ancestry estimation

We isolated DNA from peripheral blood mononuclear cells and employed a panel of 105 single nucleotide polymorphisms (SNPs) as ancestral informative markers to assess proportions of WAA, European, and Native American (NATAM) genetic ancestry.^25^ The SNPs were genotyped using the Agena MassARRAY genotyping platform according to the manufacturer’s recommendations (Agena Bioscience, San Diego, CA). Individual admixture estimates for each sample were calculated using a model-based clustering method implemented using the programme STRUCTURE v2.1.^26^ STRUCTURE 2.3 was run using *k* = 3 parental population genotypes from West Africans, Europeans, and NATAM ancestry under the Admixture model using the Bayesian Markov chain Monte Carlo method and a burn-in length of 30 000 for 70 000 repetitions. In our study, we refer to ‘ancestry’ as the estimates reported as a percentage of global ancestries for WAA, European and NATAM (combined they equal 100%).

### Flow cytometry

We stained samples according to standard protocol using the following antibodies: CD3 (clone UCHT1); CD4 (clone OKT4); CD19 (clone HIB19); CD27 (clone M-T271); CD38 (clone HIT2); CD138 (clone MI15); IgD (clone IA6-2); IgM (clone MHM-88); CXCR5 (clone J252D4); and CD11c (clone Bu15). Lymphocyte subsets: Total B cells, CD19+; Total T cells, CD3+; class-switched (CS) memory, CD19^+^ CD27^+^ IgD^−^; unswitched memory, CD19^+^ CD27^+^ IgD^+^; plasmablasts CD19^+^ CD27^hi^, CD38^+^; CS plasmablasts, CD19^+^ CD27^hi^ CD38^+^ IgD^−^; unswitched plasmablasts, CD19^+^ CD27^hi^ CD38^+^ IgD^+^; CD138 CS plasmablasts, CD19^+^ CD27^hi^ IgD^−^ CD38^+^ CD138^+^; unswitched plasmablasts, CD19^+^ CD27^hi^ IgM^+^ CD38^+^ CD138^+^; double negative (DN) B cells CD19^+^ CD27^−^ IgD^−^; DN1 B cells CD19^+^ CD27^−^ IgD^−^ CXCR5^+^ CD11c^−^; DN2 B cells CD19^+^ CD27^−^ IgD^−^ CXCR5^−^ CD11c^+^. Cell subset frequencies and counts were determined from flow cytometry data: frequencies represent the proportion of a particular gated subset among a parent population. Cell counts represent the number of events within a particular gate. Unless indicated otherwise, each data point represents the means of two, and up to five, SD for an individual research participant. Each sample drawn was separated by at least a month from other draws collected from the individual research participant.

### IgG purification from plasma

To purify bulk immunoglobulin G (IgG) from plasma samples, we employed protein G chromatography (Cytiva Life Sciences). We quantified pure IgG preparations with IgG enzyme-linked immunosorbent assay (ELISA) to facilitate normalization across all samples for assays.

### Immunocytochemistry

SH-SY5Y human neuroblastoma cells (American Type Culture Collection) were maintained according to the manufacturer’s recommendations, for immunocytochemistry experiments, cells were passaged and allowed to rest on coverslips coated with 50 µg/mL laminin (L2020, Sigma) overnight. The next day, cells were fixed with 4% paraformaldehyde (PFA; Fisher) for 10 min on ice, then washed with phosphate buffered saline (PBS) for 5 min. Cells were permeabilized with 0.2% Triton X-100 + 2 mg/mL (Sigma) for 10 min, then blocked with 0.1% Triton X-100 + 1% Goat Serum + 3% bovine serum albumin (BSA) for 2 hr at room temp. Cells were incubated with primary antibodies in a blocking buffer at 4°C overnight: 1/100 Mouse anti-microtubule-associated protein 2 (MAP2; MA5-12826, Invitrogen), 20 µg/mL purified human IgG. The next day, cells were rinsed 4 × 3 min with 0.05% Triton X-100 + 1% Goat Serum + 1% BSA, then incubated with secondary antibodies in blocking buffer at room temp for 1 hr: 1/100 Goat anti-Human conjugated AlexaFluor 488 (A11013, Life Technologies), 1/500 Goat anti-Mouse conjugated AlexaFluor 647 (ab150115, Abcam). The rinses were repeated, then cells were counterstained with 1 µg/mL 4',6-diamidino-2-phenylindole (DAPI) (62248, ThermoFisher) for 5 min. Cells were washed 2 × 5 min with PBS, then coverslips were mounted on glass slides with FluoroMount G.

### Tissue immunofluorescence

We performed immunofluorescent staining of sagittal-murine brain sections derived from experimental autoimmune encephalomyelitis (EAE)-induced mice as previously described.^27^ Briefly, diseased C57BL6/J mouse brains were obtained at the height of the disease (∼13–14 days after induction) after transcardial perfusion with PBS and 4% PFA. Brains were embedded in paraffin and 5 µm sections were taken by the UT Southwestern Histo Pathology Core.

Immunofluorescence was performed as follows: deparaffination and rehydration by 2 × 10 min washes in xylenes (Fisher), 2 × 3 min washes in 100% ethanol (Fisher), followed by 3 min washes in 95%, 70%, 50%, 30% ethanol, and PBS. Slides were washed in 15 min in 1% Glycine (Sigma) in PBS. Slides were immersed in a boiling Antigen Unmasking Solution (H-3300, Vector Laboratories) in a pressure cooker, followed by 2 × 5 min washes in PBS and 10 min wash in 0.2% Triton X-100 (Sigma). Sections were blocked with 0.1% Triton X-100 + 5% Goat Serum (50062Z, Invitrogen) for 2 hr in a humidified chamber. Sections were incubated with primary antibodies in blocking buffer at 4°C overnight: 1/100 Rabbit anti-glial fibrillary acidic protein (GFAP ab16997, Abcam), 1/200 Mouse anti-MAP2 (MA5–12826, Invitrogen), and 10 µg/mL purified human IgG. The next day, slides were rinsed 3 × 5 min in 0.025% Triton X-100, then incubated on secondary antibodies in blocking buffer at room temp for 1 hr: 1/1000 Goat anti-Human conjugated AlexaFluor 488 (A11013, Life Technologies), 1/1000 Goat anti-Rabbit conj AlexaFluor 568 (ab175471, Abcam), 1/500 Goat anti-Mouse conj AlexaFluor 647 (ab150115, Abcam). Rinses were repeated, then slices were counterstained with 1 µg/mL DAPI (62248, ThermoFisher). Slides were washed with PBS, then washed in 0.1% Sudan Black B (Sigma) in 70% ethanol for 20 min. Slides were washed 4 × in rinsing buffer, then coverslips were mounted with FluoroMount G (0100-01, SouthernBiotech). Slides were visualized on a Zeiss LSM 780 confocal microscope using a 40×/1.4 NA oil objective lens. Binding to astrocytes and neurons was determined by two independent observers blinded to the identity of the IgGs.

### Image acquisition and analysis

Images for immunocytochemistry were acquired on an inverted Nikon TI2-E CSU-W1 spinning disk confocal with a Nikon 40 × 0.95NA objective, coupled to the Hamamatsu Orca-Fusion Gen-III scientific complementary metal-oxide-semiconductor (sCMOS) camera. For each condition, we obtained 10–14 images. An average of 122 cells was quantified per image using a custom-written Fiji macro. In brief, nuclei were isolated and counted based on the DAPI channel. Then, anti-MAP2 Alexaflour 647, and anti-human IgG Alexafluor 488 signals were combined and used to determine cell masks. Next, the cytoplasmic mask was determined by removing the nuclear mask from the cell mask. Finally, the mean fluorescent intensity of anti-human IgG Alexafluor 488 was determined within the nuclear and cytoplasmic masks and plotted as bar graphs.

### Statistical analysis

When examining stratified cohorts for significant differences between outcome measures, we employed a two-tailed Wilcoxon ranked sum test (*α= 0.05*). To determine correlative relationships between genetic ancestry and outcome measures, we employed a two-tailed Spearman analysis (*α = 0.05*). We used *χ*^2^ test to determine differences between IgG samples containing neuron, or astrocyte-binding antibodies. We employed robust regression and outlier removal (ROUT) analysis (*Q= 1%*) to account for outliers in our categorical cohort data. We further constructed multivariable linear regression models comparing WAA ancestry with either disease duration; number of NAT infusions; or body mass index (BMI). Models included interaction terms fit according to ordinary least squares, and two-sided *P* values are reported. *P* values <0.05 were considered statistically significant.

## Results

### Study population and ethnicity-stratified cohorts

To determine the relationship between self-reported ethnicity, genetic ancestry and measures of T cell-dependent humoral B cell responses in the context of MS, we conducted flow cytometric analysis on fresh peripheral blood mononuclear cells obtained from repeat blood SD between 2019 and 2021 (BA *= 2.5* and W *= 2.4* average SD: 1.2; 1.1, *P = 0.65*)(Table 1). Our sample entailed 67 consenting participants with relapsing-remitting MS (MS) managed on monthly NAT infusion therapy.

**Table 1:** Summary of study population demographics

Ethnicity Cohort	BA	W	*P* values	LA (neither BA, nor W)
*n*	26	36		5
Mean age (SD)	37.5 (10.7)	38.2 (9.7)	ns (*P= 0.73*)	35.8 (4.2)
Sex (F:M)	5.5 (22:4)	3.5 (28:8)	ns (*P* = 0.85)	(5:0)
Disease duration (SD)	76.4 (51.06)	88.8 (73.5)		93 (62.8)
No. NAT infusions (SD)	45.3 (38.8)	58.6 (40.8)	ns (*P= 0.14*)	42.8 (21)
BMI (SD)	29.8 (8.5)	25.4 (5.6)	* (*P= 0.03*)	29.7 (1.8)
*n* with ancestry estimate data	22	10		3
%WAA	72 (11)	6 (8)	* (*P* < *0.0001*)	42 (14)
%European	20 (13)	86 (9)	* (*P* < *0.0001*)	53 (8)
%NATAM	8 (5)	8 (4)	ns (*P= 0.89*)	5 (5)
Mean no. SD	2.5 (1.2)	2.4 (1.1)	ns (*P= 0.65*)	2.4 (0.8)

For categorical analysis, we compared two primary cohorts stratified based on self-reported ethnicity into: BA (*n = 26*), and, White (W, *n = 36*). The two cohorts were age-matched (BA = 37.5, W = 38.2, *P = 0.73*), and had a skewed female-to-male ratio (BA = 5.5, W = 3.5). Despite skewed sex ratios, we found that significant differences between BA and W cohorts remained even when analysed among female participants. Similarly, there was no significant difference in disease duration (as defined as the months between diagnosis to the first sample draw of this study) between ethnicity cohorts (BA = 76.4, W = 88.8, *P = 0.85*). There was also no difference between BA and W cohorts in the time between diagnosis and initiation of disease-modifying therapy (not shown). Participants in the W cohort had an overall greater (but non-significant, *P = 0.14*) number of total NAT infusions. There was a modest but significant difference in BMI between the two study cohorts (BA = 29.8, W = 25.4, *P = 0.03*). (Table 1) We determined proportions of WAA, European (EURO) and NATAM ancestry estimates for a subset (*n = 35*) of our study population across all ethnicity cohorts (BA, *n = 22*; W, *n = 10*; LA, *n = 3*). Compared to the overall study population, the ancestry subset was matched in age, number of NAT infusions, and had a similarly skewed female-to-male ratio (data not shown).

### Ethnicity-, and ancestry-associated lymphocyte dynamics in multiple sclerosis

B and T lymphocytes work in concert to generate effective and targeted humoral immunity. In the context of MS, these cells play a prominent role in acute clinical relapse and radiological lesion manifestations. Heightened severity among BA and Latin American patients may result from ethnicity-associated differential frequencies of immunopathic lymphocytes. However, published data on potential ethnicity-associated immunologic trends in MS are scarce, with minimal lymphocyte subset data reported to date.^22^

**Figure 1:**
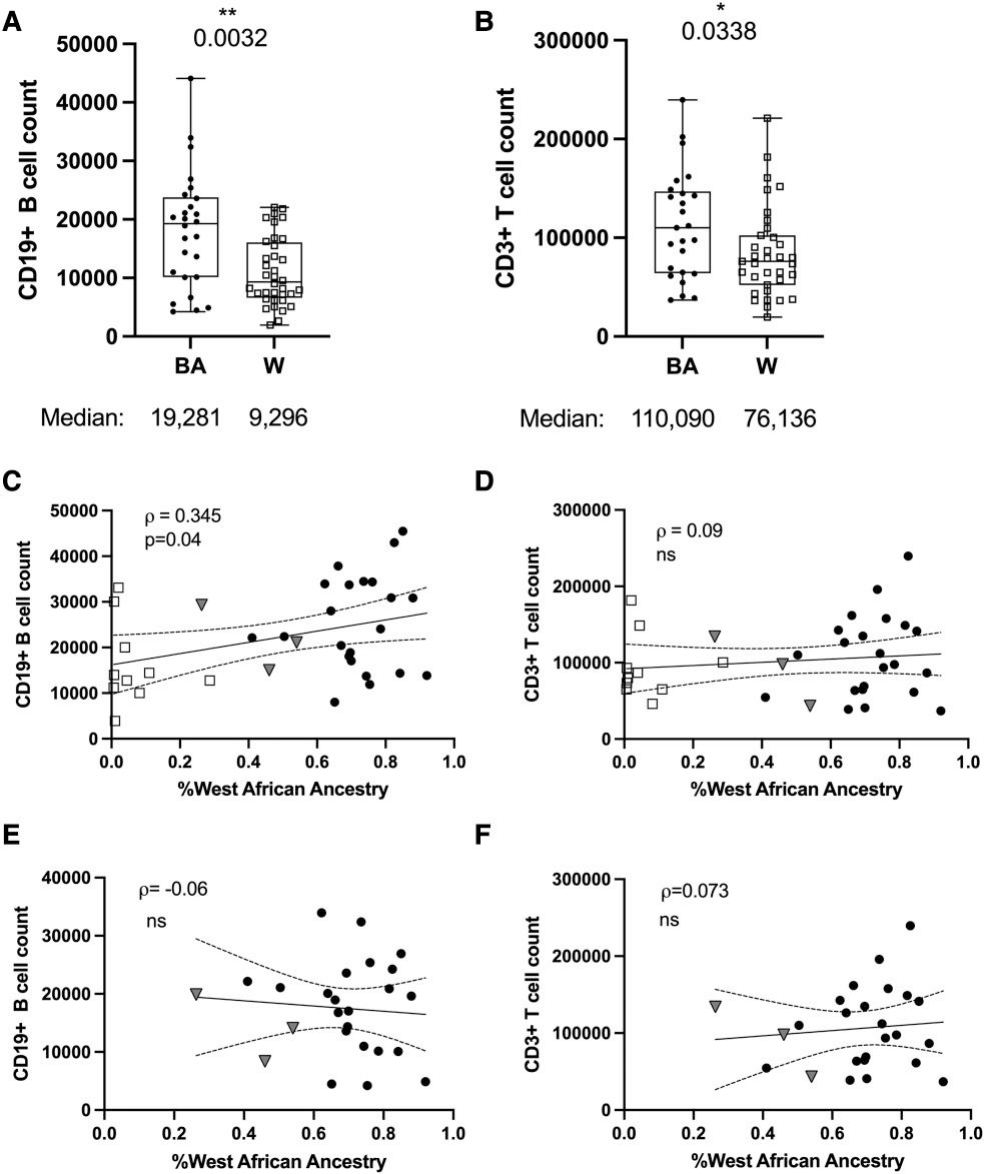
**Heightened levels of B cells and T cells are associated with BA ethnicity and not WAA genetic ancestry**. Box plots displaying (**A**) absolute CD19+ B cell and (**B**) CD3+ T cells counts within BA, and White (W) Cohorts. Correlations between absolute B (**C**) and T cell (**D**) counts and the percentage of genetic WAA ancestry across all study participants for which genetic ancestry data was collected, or (**E, F**) among participants identifying with BA, or only Latin American ethnicity. BA = full circles; White (W) = EMPTY squares; Latin American = grey triangles. Box plots display median summary values; each data point represents the mean of two-to-five sample observations for an individual research participant; error bars represent the range. Significance determined by Wilcoxon ranked sum test (**A, B**), and Spearman’s correlations (**C-F**). Two-sided *P* values are reported, and *P* values <0.05 were considered statistically significant.

To test our hypothesis that levels of WAA ancestry would correlate with differential T-dependent B lymphocyte dynamics across categorical ethnicity, we examined the relationship between genetic ancestry and lymphocyte metrics in our ethnicity-stratified cohorts. Study cohort participants were on the therapy NAT, which effectively sequesters circulating B and T cells in the periphery, limiting their capacity to cross the blood-brain barrier. This enables analysis of lymphocytes (including potentially immunopathic B and T cells), that may have otherwise migrated into the CNS. The BA cohort exhibited twice the number of circulating B cells compared to White-identifying participants (*P = 0.003*; Fig. 1A). Alongside this stark B cell differential, we also observed a 44% increase of total T cells in the BA cohort compared to White-identifying participants (*P = 0.03*; Fig. 1B). When examining the frequencies of B cells and T cells among total lymphocytes we observed no differences in the cohorts (Supplementary Fig. 1).

When analysing the total study sample by WAA ancestry across all categorical ethnicity groups, we found a significant positive correlation between WAA ancestry and the level of CD19^+^ B cells, (*P = 0.04*; Fig. 1C) but not CD3^+^ T cells (*P = 0.57*; Fig. 1D). WAA ancestry is relatively negligible among individuals identifying with White categorical ethnicity, (Table 1) but enriched to varying levels among those identifying with the BA or Latin American diasporas.^28^ We, therefore, examined the potential for correlative relationships between WAA ancestry and B- or T-cells among individuals identifying with BA, or LA/Hispanic ethnicity. Through this sub-analysis, we observed that there was similarly no notable relationship between genetic WAA ancestry and either lymphocyte population among participants identifying with the Black and/or LA/Hispanic ethnicity [*P = 0.76*; Fig. 1E, (*P = 0.72*); Fig. 1F].

### Ethnicity-, and ancestry-associated memory B cell subset differentials in multiple sclerosis

The heightened levels of both total B cells and T cells in our BA cohort (see Fig. 1A and B), and the primary role T cells play in the differentiation of memory B cells into class-switched subtypes, prompted a closer investigation of B cell subsets. We observed a significantly reduced frequency of total memory B cells of CD19^+^ B cells among W compared to the BA cohorts (*P < 0.003*). However, there was no significant difference in the total number of memory B cells between the cohorts (Supplementary Fig. 1). Clonal analysis of B cell receptor repertoires suggest CS memory B cells represent the bulk of immunopathic B cells trafficking from the blood into the CNS during MS.^15,29^ We delineated memory B cells according to the presence of CD27, and surface IgD, into class switched (IgD^−^) and unswitched (IgD^+^) subsets. Applying a phenotyping strategy based on these markers, we quantified absolute counts and frequencies of circulating CS and unswitched (Usw) memory B cells within the total CD19^+^ B cell pool.

**Figure 2:**
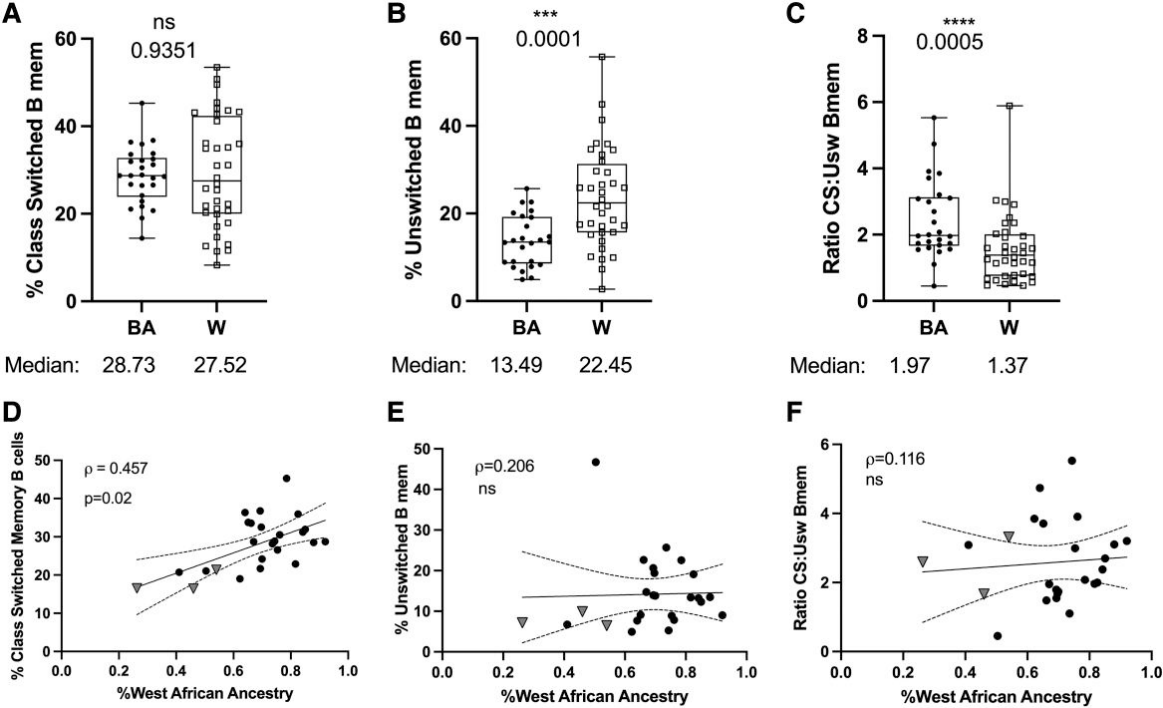
**The frequency of CS memory B cells is not associated with BA ethnicity but significantly correlated with WAA ancestry**. Flow cytometry plots displaying frequencies and ratios of CS and unswitched memory B cells within BA and White cohorts, and according to WAA ancestry among participants identifying with BA or only Latin American ethnicity. (**A**) Association between self-identified ethnicity and percent frequencies of CD19^+^ CD27^+^ IgD^−^ CS B cells, CD19^+^ CD27^+^ IgD^+^ unswitched B cells, or the ratio of CS and unswitched B cell counts. (**B**) Correlations between WAA ancestry and CD19^+^ CD27^+^ IgD^−^ CS B cells, CD19^+^ CD27^+^ IgD^+^ unswitched B cells, or the ratio of CS and unswitched B cell counts among participants identifying with BA, or Latin American ethnicity only. BA = Full circles; White (W) = **E**mpty squares; Latin American = grey triangles. Box plots display median summary values; error bars represent range; each data point represents the mean of two-to-five sample observations for an individual research participant. Significance determined by Wilcoxon ranked sum test (**A–C**), and Spearman’s correlations (**D–F**). Two-sided *P* values are reported, and *P* values <0.05 were considered statistically significant.

We found that the frequencies of CS memory B cells were equivalent between BA and W cohorts (Fig. 2A). By contrast, unswitched memory B cells were almost twice as frequent in the W cohort compared to the BA cohort (*P < 0.0001*; Fig. 2B). CS memory B cells matriculate from a pool of unswitched memory B cells, which, upon switching antibody class, irreversibly lose their ability to express IgD. To better understand class switch recombination dynamics, we expressed the quantities of CS and unswitched memory B cells as the ratio of CS-to-unswitched memory B cells. Compared to our W cohort, the BA cohort exhibited a significantly greater ratio of CS-to-unswitched memory B cells (*P < 0.0005*; Fig. 2C). When assessing the BA cohort for a relationship between WAA ancestry and memory B cell metrics, we found a positive correlation between WAA ancestry, and the frequency of CS memory B cells (*P = 0.02*; Fig. 2D). However, there was no correlation between levels of WAA ancestry and frequencies of unswitched memory B cells, or CS:Usw ratio (*P = 0.32*; Fig. 2E, *P = 0.57*; Fig. 2F).

### Double negative 2 B cells, ethnicity, and ancestry in multiple sclerosis

**Figure 3:**
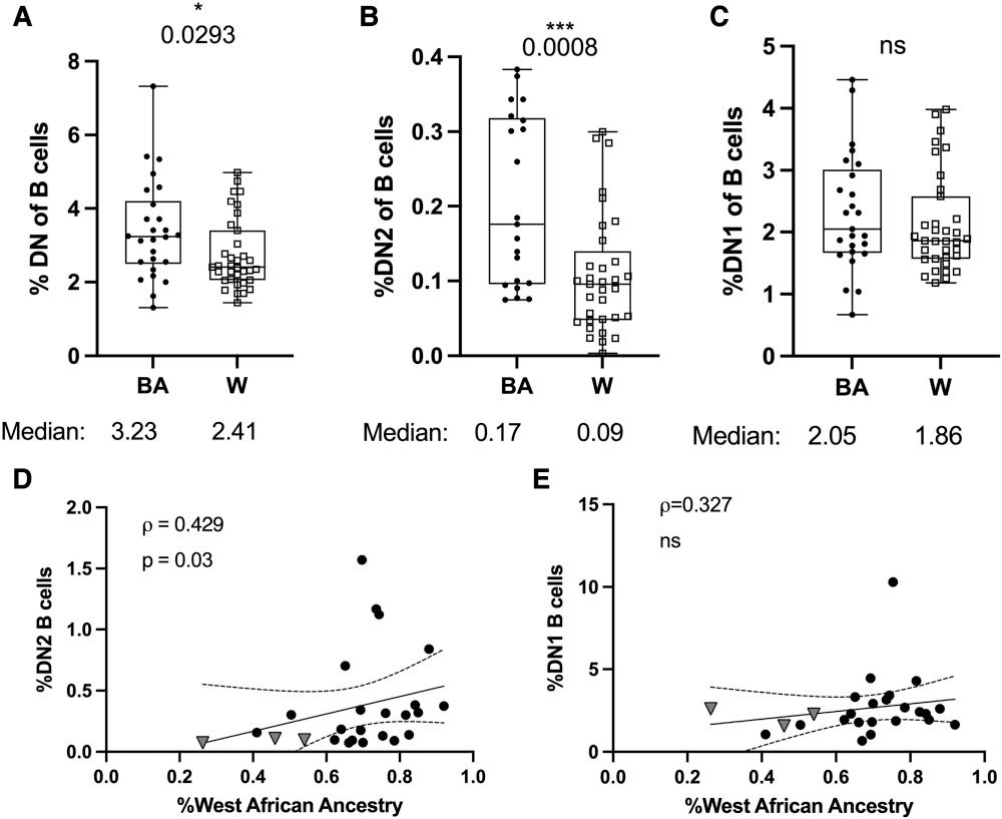
**DN 2 B cells are associated with self-identified BA ethnicity and positively correlated with WAA genetic ancestry**. Box plots displaying frequencies of (**A**) total DN, as well as (**B**) DN2, and (**C**) DN1 subsets for BA, and White cohorts. Correlations between the percent frequencies of (**D**) DN2 and (**E**) DN1 B cells among total CD19+ B cells and genetic WAA ancestry among participants identifying with BA, or only Latin American ethnicity. BA = Full circles; White (W) = Empty squares; Latin American = grey triangles. Box plots display median summary values; error bars represent range; each data point represents the mean of two-to-five sample observations for an individual research participant. Significance determined by Wilcoxon ranked sum test (**A–C**), and Spearman’s correlations (**D, E**). Two-sided *P* values are reported, and *P* values <0.05 were considered statistically significant.

Double negative (DN) B cells are a recently identified antigen-experienced B cell subset expressing antibody rearrangements with somatic hypermutation accumulation that is increased intrathecally during acute MS disease.^15,30^ DN2 B cells, a subset of DN B cells, are established contributors to autoantibody responses and disease severity in systemic lupus erythematosus (SLE). Some reports suggest that DN2 B cells are elevated among individuals self-identifying with the BA diaspora^31,32^ In contrast to DN2 B cells, DN1 B cells decline in frequency according to SLE severity,^33^ and more closely resemble memory B cells rather than antibody-secreting cell transcriptional profiles.^32^ We examined levels of these DN B cell subsets in our study sample to determine relationships between the levels of these cells, self-identified ethnicity and WAA ancestry. The frequency of DN B cells among total B cells was significantly greater in the BA cohort relative to the W cohort (*P < 0.03*; Fig. 3A). This difference persisted in DN2 (*P < 0.0008*; Fig. 3B) but not DN1 (*P = 0.37*; Fig. 3C) B cells. Similarly, DN2 (*P = 0.03*; Fig. 3D) but not DN1 (*P = 0.13*; Fig. 3E) B cells exhibited a significant positive correlation with WAA ancestry within the BA cohort.

Ultimately, effective humoral immune responses require B cells to differentiate into ASCs. ASCs are the principal source of immunoglobulins (antibodies) and represent a terminal B cell effector state. Several studies link greater ASC levels or immunoglobulin production with both acute inflammatory, and chronic degenerative aspects of MS. Notably, intrathecal ASC activity appears to strongly correlate with greater grey matter atrophy observed among those identifying with BA ethnicity.^17^ Our prior cross-sectional study demonstrated that circulating plasmablasts (an ASC subtype) were significantly heightened among Black, or Latin American-identifying study participants with MS relative to those identifying as White.^22^

To expand upon these findings, we quantified average frequencies and counts of several plasmablast subtypes over multiple SD and in relation to WAA genetic ancestry across ethnicity categories. The frequency of total CD27^hi^ CD38^+^ plasmablasts was similar between the BA and W cohorts, while an overall number of total plasmablasts was significantly increased among the BA, compared to the W cohort (*P = 0.0004;*Supplementary Fig. 1).

**Figure 4:**
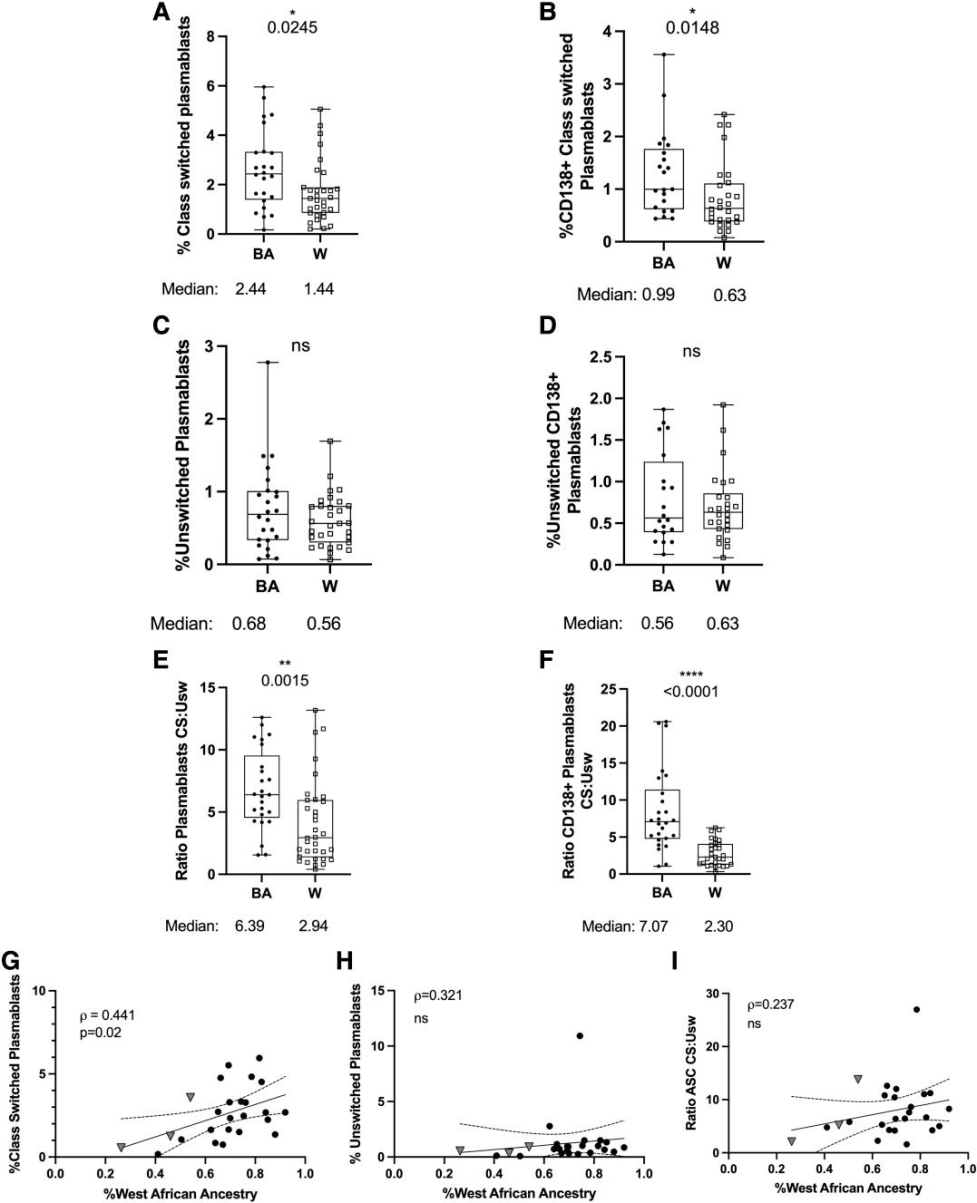
**The frequencies of CS ASC are associated with BA ethnicity, and positively correlated with WAA ancestry**. Flow cytometry plots displaying frequencies and ratios of CS and unswitched plasmablasts within BA, and White cohorts, and according to WAA ancestry among participants identifying with BA, or Latin American ethnicity only. Displayed are the association between self-identified ethnicity and (**A**) percent frequencies of CS CD27^hi^ CD38^+^ plasmablasts, (**B**) CS CD138^+^ plasmablasts; (**C**) unswitched CD27^hi^ CD38^+^ plasmablasts, (**D**) unswitched CD138^+^ plasmablasts (**E**) the ratio of CS-to-unswitched CD27^hi^ CD38^+^, or (**F**) ratio of CS-to-unswitched CD138^+^ plasmablasts. (**G**) Correlations between WAA ancestry and CD27^hi^ CD38^+^ plasmablasts, (**H**) unswitched CD27^hi^ CD38^+^ plasmablasts, or (**I**) the ratio of CS and unswitched CD27^hi^ CD38^+^ plasmablasts among participants identifying with BA, or only Latin American ethnicity. BA = full circles; White (W) = empty squares; Latin American = grey triangles. Box plots display median summary values; error bars display range; each data point represents the mean of two-to-five sample observations for an individual research participant. Significance determined by Wilcoxon ranked sum test (**A–F**), and Spearman’s correlations (**G–I**). Two-sided *P* values are reported, and *P* values <0.05 were considered statistically significant.

In addition to governing memory B cell differentiation, T cells also play a primary role in the differentiation of class-switched plasmablasts. We, therefore, examined the ratios of CS-to-unswitched plasmablasts among our cohorts. Average frequencies of CS CD27^hi^ CD38^+^, (*P = 0.02*; Fig. 4A) and CS CD138^+^ (*P = 0.01*; Fig. 4B) plasmablasts were significantly increased among the BA cohort relative to the W cohort. The difference in CS CD27^hi^ CD38^+^ plasmablasts was also increased for the BA cohort when examined as three individual timepoints (Supplementary Fig. 2). We did not observe any difference among the frequencies of unswitched CD27^hi^ CD38^+^, (*P = 0.37*; Fig. 4C) or CD138^+^ (*P = 0.92*; Fig. 4D) plasmablasts between the cohorts.

There was a significantly greater CS-to-unswitched plasmablast ratio, both for CD27^hi^ CD38^+^ (*P = 0.0002*; Fig. 4E) as well as CD138^+^ (*P < 0.0001*; Fig. 4F) subpopulations in the BA, compared to W cohorts. Of these metrics, only the frequency of class-switched CD27^hi^ CD38^+^ plasmablasts (*P = 0.02*; Fig. 4G) exhibited a significant positive correlation with WAA ancestry (*P = 0.11*; Fig. 4H, *P* = 0.25; Fig. 4I).

### Neuronal-binding antibodies, ethnicity, and ancestry in multiple sclerosis

MS is characterized by the production of autoreactive antibodies that bind to a range of CNS targets. These antigens may be present in both white and grey matter and found on the surface, or intracellularly among neurons or glial cells. Autoreactive antibodies capable of promoting demyelinating activity may be found in circulation^34^ as well as in the CSF.^35,36^ This parallels the trafficking patterns of pathogenic B cells and ASCs into the CNS. Whether antibodies in MS exhibit differential CNS antigen autoreactivity among BA-, or White-identifying individuals with MS is unreported in the literature.

**Figure 5:**
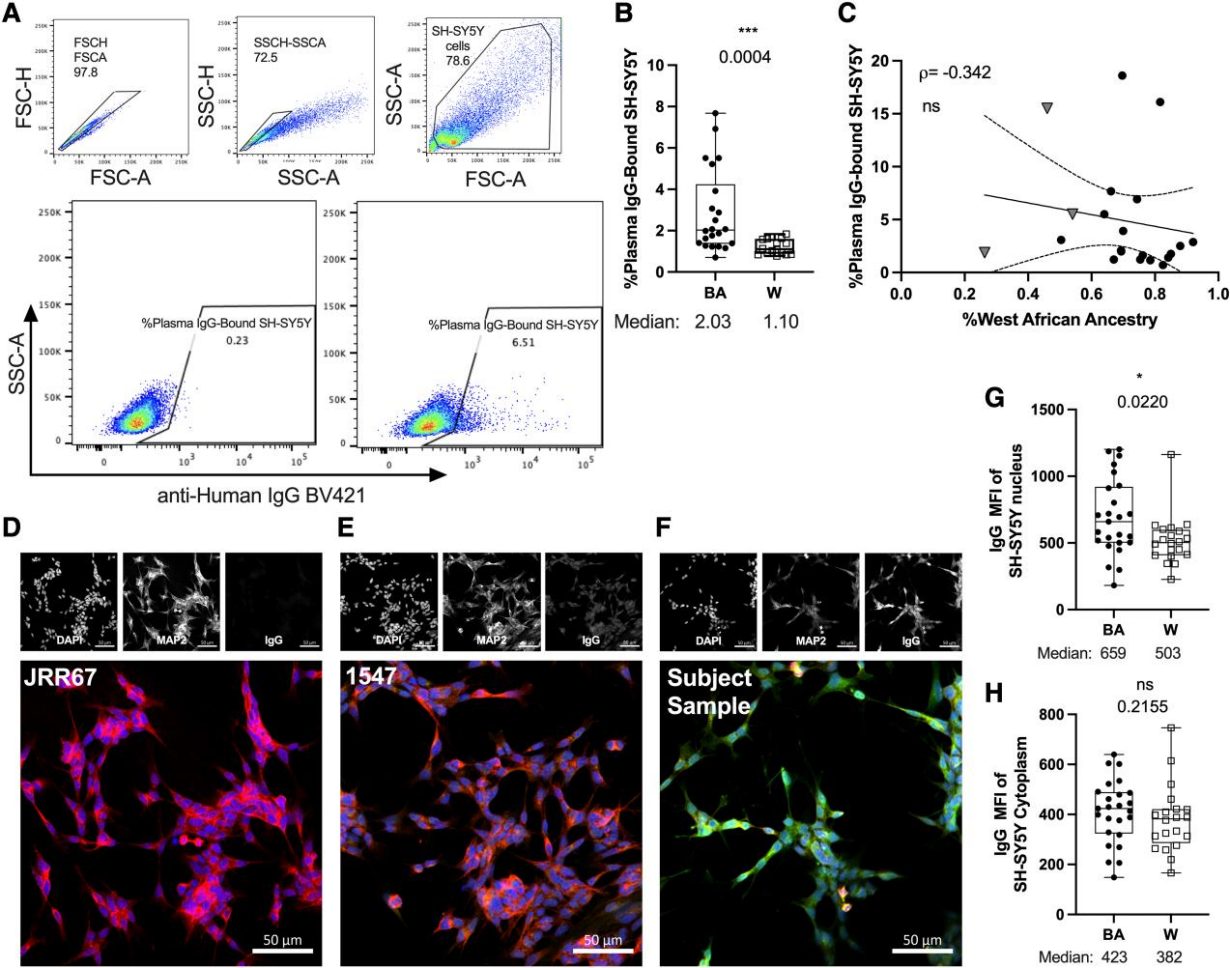
**SH-Sy5y-binding antibody levels are associated with BA ethnicity, and not correlated with WAA ancestry**. Box plots depict SH-SY5Y cells surface-stained with purified plasma-IgG (**A**–**C**), or after fixation/and permeabilization visualized with immunocytochemistry (**D**–**H**). Representative flow cytometry gating strategy showing doublet and debris exclusion (upper panels), and sample plots of unstained (**A**, lower left panel), as well as anti-human IgG positively stained cells (**A**, lower right panel). (**B**) Quantification of the percent frequency of SH- SY5Y cells positive for anti-human IgG staining for BA, and White cohorts. (**C**) Correlation between WAA ancestry and percent frequency of SH-SY5Y cells positive for anti-human IgG staining among participants identifying with BA, or only Latin American ethnicity. (**D**–**F**) Sample plots depicting SH-SY5Y cells stained with various patient-derived IgG: (**D**) monoclonal IgG antibody with no reactivity to neurons;^37^ (**E**) Purified plasma bulk-IgG derived from a patient with N-methyl-D-asparatate receptor (NMDAR)-encephalitis; (**F**) Purified plasma bulk-IgG derived from a subject within the study cohort. Human IgG (Alexafluor 488); MAP2 (Alexafluor 647); Nuclei (DAPI). Image scale bars represent 50 µm. Mean fluorescence intensity of IgG-bound to SH-SY5Y nuclei (**G**), and cytoplasm (**H**) within BA, and White cohorts. BA = Full circles; White (W) = Empty squares; Latin American = grey triangles. Box plots display median summary values; error bars display range; each data point represents an INDIVIDUAL research participant. Significance determined by Wilcoxon ranked sum test (**B, G, H**), and Spearman’s correlations (**C**). Two-sided *P* values are reported, and *P* values <0.05 were considered statistically significant.

To examine the relationships between CNS antigen reactivity and self-reported ethnicity, or genetic ancestry, we purified total circulating IgG from plasma samples collected during sample blood draws. First, we employed purified total IgG in assays involving the neuroblastoma cell line SH-SY5Y. Upon surface staining SH-SY5Y cells with purified plasma-IgG (Fig. 5A), we observed a significantly greater frequency of IgG-positive SH-SY5Y cells among the BA cohort, compared to the W cohort (*P = 0.0004*; Fig. 5B). The frequency of IgG-positive SH-SY5Y cells exhibited a negative, non-significant correlation with WAA ancestry (*P = 0.12*; Fig. 5C). We subsequently visualized SH-SY5Y cells by fluorescent microscope after fixation and permeabilization. Purified IgG exhibited notable binding throughout the cytoplasm and nucleus among fixed and permeabilized SH-SY5Y cells relative to negative and positive controls (Fig. 5D–F). Nuclear IgG staining was significantly more pronounced among the BA cohort, compared to the W cohort (*P = 0.02*; Fig. 5G). By contrast, there was no significant difference in cytoplasm IgG staining between ethnicity cohorts (*P = 0.21*; Fig. 5H). Neither nuclear nor cytoplasm-associated IgG mean fluorescence intensity correlated with WAA ancestry (Not shown).

To determine the relationship between self-reported ethnicity and relative levels of brain tissue reactive IgG, we incubated purified total IgG from 24 subject samples (Supplementary Fig. 3) with brain sections obtained from EAE-induced mice. Subject samples were selected from each of the BA (*n* = 12) and W (*n* = 12) cohorts from those exhibiting the greatest (*n* = 6) and lowest (*n* = 6) frequencies of switched plasmablasts. We used EAE tissue as the inflammatory environment evoked in the EAE model more accurately reflects the generalized inflammation observed in MS CNS tissue.

We subsequently assessed tissue for neuronal or astrocyte binding based on staining fluorescent patterns between IgG, and MAP2, or GFAP. Staining pattern analysis was conducted by two independent analysts who were blinded to IgG sample subject data. IgG-bound tissue displayed several neuronal, and/or astrocyte staining patterns: ranging from the absence of IgG signal; to compartmentalized nuclear-, or cytoplasmic binding; or more diffuse staining throughout granular or hilar neuronal bodies.

**Figure 6:**
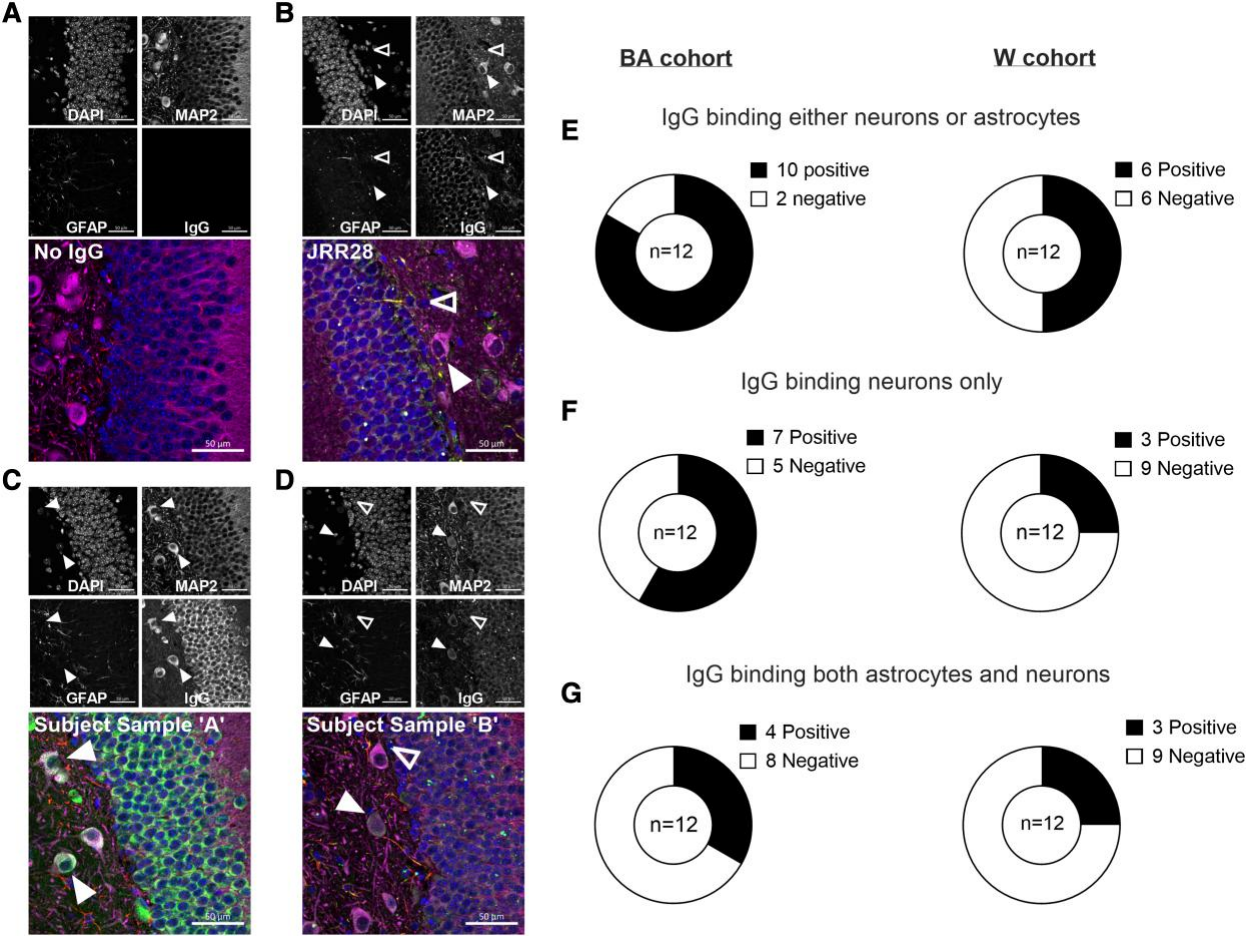
**BA ethnicity is associated with a greater frequency of neuron-binding IgG**. Representative images of sagittal brain sections from EAE-induced mice stained (**A**) without subject sample IgG; (**B**) with a positive control IgG (Alexafluor 488) exhibiting MAP2 (Alexafluor 647)-expressing neuronal, as well as, GFAP^+^ astrocyte (Alexafluor 568) binding; (**C**) subject sample IgG exhibiting strong binding to neurons only. (**D**) Subject sample IgG exhibiting neuron and astrocyte staining. Comparisons between categorical ethnicity cohorts, (BA, *n* = 12; **W**, *n* = 12) of IgG samples exhibiting (**E**) positive staining for either neurons or astrocytes (χ^2^ = 3, d.f. = 1, *P* = 0.08), (**F**) staining of neurons and not astrocytes (χ^2^ = 2.74, d.f. = 1, *P* = 0.09), or (**G**) dual binding of neurons and astrocytes (χ^2^ = 0.2, d.f. = 1, *P* = 0.65). MS patient-derived IgG monoclonal antibodies served as neuron-& astrocyte-positive (**B**) antibody staining control. Closed arrows point to examples of IgG-stained neurons and open arrows point to IgG-stained astrocytes. (Image scale bars represent 50 µm. Donut charts display the proportion of neuron- or astrocyte-binding antibodies. Significance level derived from *χ*^2^ test (Panels **E–G**). Two-sided *P* values are reported, and *P* values <0.05 were considered statistically significant.

IgG samples from our BA cohort exhibited greater overall positive binding to brain neuron or astrocyte populations with 83% positive IgG samples (10 of 12) among our BA cohort compared to 50% (6 of 12) among our W cohort (χ^2^ (*1, n = 24*)*= 3*, *P = 0.08;*Fig. 6E). Our BA cohort exhibited 2.32-fold the frequency of IgG that bound only to neurons, and not astrocytes 58% (7 of 12), relative to our W cohort 25% (3 of 12) (χ^2^ (*1, n = 24)= 2.7*, *P = 0.09*; Fig. 6F). By contrast, 33% (4 of 12), compared to 25% (3 of 12) of IgG samples exhibit neuron and astrocyte-binding staining patterns (χ^2^ (*1, n = 24)= 0.2*, *P = 0.65*; Fig. 6G). No notable differences in binding patterns were found according to high or low switched plasmablast frequencies, neither within, nor between cohorts.

## Discussion

The overall finding of this study is that during MS, BA ethnicity is associated with heightened T cell-dependent neuron-reactive antibody responses relative to White-identifying MS patients.

Ethnicity-associated disparity in studies of MS consists largely of work examining differential clinical presentation, and MS incidence. These investigations demonstrate that self-reported ethnicity labels reveal differential MS incidence risk alleles,^38,39^ onset kinetics,^9^ and consistently more severe clinical^2,4,6,40^ as well as paraclinical^3,41^ presentation among those identifying with BA/African American, or LA/Hispanic ethnicity. Self-reported categorical ethnicity labels may also distinguish immunological features, as demonstrated by our own prior work,^22^ and that of others.^17,20,21,42^ These studies point to a potential role for IgG-producing ASCs^19^ in contributing to ethnicity-associated clinical discordance in MS.

Contrasting our prior cross-sectional approach, we leveraged multiple SD to determine the durability of differential ethnicity-associated ASC frequencies, and to quantify lymphocyte populations implicated in antibody generation as well as MS pathogenesis. Flow cytometry data for each study participant in our present study reflects the mean of two, and up to five, SD, with each draw separated by at least a month. Mean data comprised all sample draw data collected for each study participant. Our data therefore demonstrates that differential ASC frequencies are sustained over time (Supplementary Fig. 2). Further, we show that this ethnicity-associated ASC differential parallels significantly heightened levels of CS memory B cells, and DN B cells; two B cell subpopulations that are both implicated in MS disease severity,^15,29^ and contain key upstream ‘pre-ASC’ B cells prone to differentiation into IgG-producing ASCs.^32,43^

Future work involving BA ethnicity-associated immunobiology during MS should include DN2 B cells in their analyses. In this study, we show that during MS heightened DN2, and not DN1 B cell levels are associated with BA ethnicity. While the involvement of DN2 B cells in MS pathogenesis is currently unspecified, DN2 B cells play prominent roles in SLE^32,44^ and COVID19^45^ disease activity among African American patients. In the context of MS, several studies show that the broader DN B cell population (of which the DN2 B cells are a subset) is increased in circulation,^30,46^ and elevated in the CNS during acute gadolinium-enhancing lesion activity.^15^ Moreover, DN B cells, as well as related upstream populations from which DN2 B cells derive^32^ are associated with the production of autoreactive antibodies during SLE,^32,44^ as well as other autoimmune or infectious neurological conditions.^30^ The precise identity and relevance of DN subsets other than DN1 and DN2 remain to be specified in MS and autoimmunity. Ultimately, delineating the contributions of specific B cell subsets, such as DN2 B cells, from within larger populations may present an opportunity for more precise therapeutic development beyond broad lymphocyte depletion.

We show an association between self-reported BA ethnicity and a significantly elevated prevalence of BA subjects with neuronal-binding IgG compared to self-reported White ethnicity. CNS-autoreactive antibodies are increased in individuals with relapsing-remitting MS.^47^ However, no study in the present literature examines how categorical self-reported ethnicity influences autoreactive antibody dynamics in MS. IgG in MS may bind neuronal, or glial targets,^37^ with some of these antibodies inducing complement-mediated demyelination or axon loss.^34‐36^ Of clinical significance, IgG production is positively associated with CNS atrophy in MS,^17^ and MS disability scores.^19^ Given the above, our finding of differential ethnicity-associated neuronal-reactivity supports the contribution of antibodies towards the greater demyelinating lesion volume^3,41^ atrophy^6,17,48^ and disability,^2,4^ measures exhibited by patients identifying with BA diasporic ethnicity. Conversely, these antibodies may possibly also function in a regulatory, neuroprotective capacity. Future work should examine ethnicity-associated differences in specific antibody target antigen, as well as antibody contribution to immunopathic or neuroprotective function.

As described previously (see Materials & Methods, Ethnicity cohort stratification) the BA cohort included participants that identified with Black ethnicity solely, as well as those identifying both with Black ethnicity as well as Latin American ethnicity. There were no changes in the significant differences between the BA and W cohorts for any measured outcomes if the BA cohort comprised participants who identified only with Black ethnicity (excluding those identifying with both Latin American ethnicity, as well as Black ethnicity).

Categorical ethnicity is ultimately constrained by its broad societally constructed application and limited suitability in stratifying multi-categorical ethnicity for research.^49^ Building upon our previous findings, we conducted the first analysis of MS B cell dynamics according to genetic ancestry. Our results show that WAA ancestry positively correlates with CS memory, DN2 and CS ASCs. European ancestry often exhibited an indirect relationship with B cell dynamics in our study (not shown). BA/African American ethnicity is enriched with high levels of WAA ancestry while admixed populations in the US exhibit variable, but substantial WAA ancestry levels. This suggests that WAA ancestry may function as an effective correlate to stratifying T cell-dependent B cell responses in future studies involving admixed populations.

Indeed, we show that WAA ancestry delineates B cell relationships among our cohort, which comprises participants with a range of admixture, including those self-identifying with Latin American ethnicity, or multiple categorical ethnicity labels. Future work employing ethnicity and ancestry may pave the way for admixture mapping approaches that allows the identification of genetic loci contributing to downstream ethnicity-associated ASC responses. Yet, while genetic ancestry positively correlated with B cell populations suggestive of heightened T cell-dependent antibody-producing B cell response, there was no significant relationship between genetic ancestry and neuron-reactive antibodies. One issue could be that the sensitivity of current approaches is insufficient to detect all neuron-reactive antibodies. This finding may also point to a range of social, behavioural or environmental, mediating factors unaccounted for in our analysis.

There are several limitations to our study. These include the use of a single site for participant recruitment, and that neither ancestry estimate data, nor plasma samples were available for all participants for the total study population. NATAM ancestry was not present in substantial quantities for most of our study population to establish appropriate correlations with outcome measures.

We conducted a regression analysis to determine the contribution of the number of NAT infusions, BMI or disease duration. We found that none of these factors significantly contributed to immunologic outcomes that separately correlated with WAA ancestry. However, these analyses are hypothesis-generating, and should be validated in larger cohorts during future work (Supplementary Table 1). There were no significant relationships between the number of NAT infusions and any of the B or T cell outcome measures among either BA or W cohorts (Data not shown).

Ethnicity-associated humoral immunologic differences observed in this study were unlikely due to the influence of NAT. While the study population was treated with NAT throughout sample collection, prior work demonstrates that treatment status does not affect ethnicity-associated differences in circulating plasmablast levels during MS,^22^ or intrathecal IgG. Further, effective humoral immunity remains intact despite NAT treatment.^24^ We collected BMI measures sporadically throughout sample collection, however, BMI data was not recorded for each sample drawn. We did not observe any notable relationship between neuronal-binding IgG and BMI.

We separately determined relationships between either self-reported ethnicity or genetic ancestry, with immunologic data. Alongside ethnicity and genetic ancestry, future studies should also include several components of social determinants of health, such as education level, income and residential zip code in conjunction with biological correlates. These collective data would enable a more comprehensive analysis of mediating and moderating effects of socially determined factors on ethnicity-associated immunobiology in MS.^8,10,50‐52^

Finally, while we do not currently have clinical or radiologic datasets to determine relationships between immunologic outcomes, ethnicity, and ancestry, these are data that will be the focus of future investigation. Future work reconciling self-reported ethnicity, genetic ancestry and social determinants of health will be key to unravelling and effectively treating the disparate disease course experienced on average by individuals of BA, and Latin American ethnicity.

Despite these limitations, our study demonstrates for the first time that genetic ancestry may be employed to identify B cell population dynamics, and neuron-reactive antibodies that may be relevant to MS immunopathogenesis in an admixed cohort. Further, it provides initial insight into the potential contribution of antibodies to ethnicity-associated MS severity discordance.

## Supplementary Material

fcad218_Supplementary_Data

## Data Availability

Data is available from the corresponding author upon request.
