# Supplementary material for: Neuron-binding antibody responses are associated with Black ethnicity in multiple sclerosis during natalizumab treatment
Source: Brain Commun. 2023 Aug 14;5(4):fcad218. doi: 10.1093/braincomms/fcad218 (PMC10433937; doi:10.1093/braincomms/fcad218)
Supplement: fcad218_Supplementary_Data [file fcad218_supplementary_data.docx]

**Supplementary material**

**Supplementary Figure 1. Quantitation of total memory B cells, plasmablasts, B and T cells**. Flow cytometry data displaying median values of the indicated measures. Each data point represents the mean of two to five sample observations for an individual research participant.

**Supplementary Figure 2.** Elevated plasmablast levels in the Black African-participant cohort sustained over three independent sample draws. Flow cytometry data displaying frequencies of class-switched plasmablasts within Black African and White cohorts (BA n=16, W (n=15, timepoint 1; n=13, timepoint 2; n=12, timepoint 3)) from three independent sample draws obtained over the course of 2 years. Displayed are the association between self-identified ethnicity and percent frequencies of class-switched CD27^hi^ CD38^+^ plasmablasts. BA=Black African; W=White cohorts. Box plots display median summary values; Error bars display range; Each data point represents an individual research participant. Significance determined by Wilcoxon ranked sum test. Two-sided p values are reported, and p values < 0.05 were considered statistically significant.

**
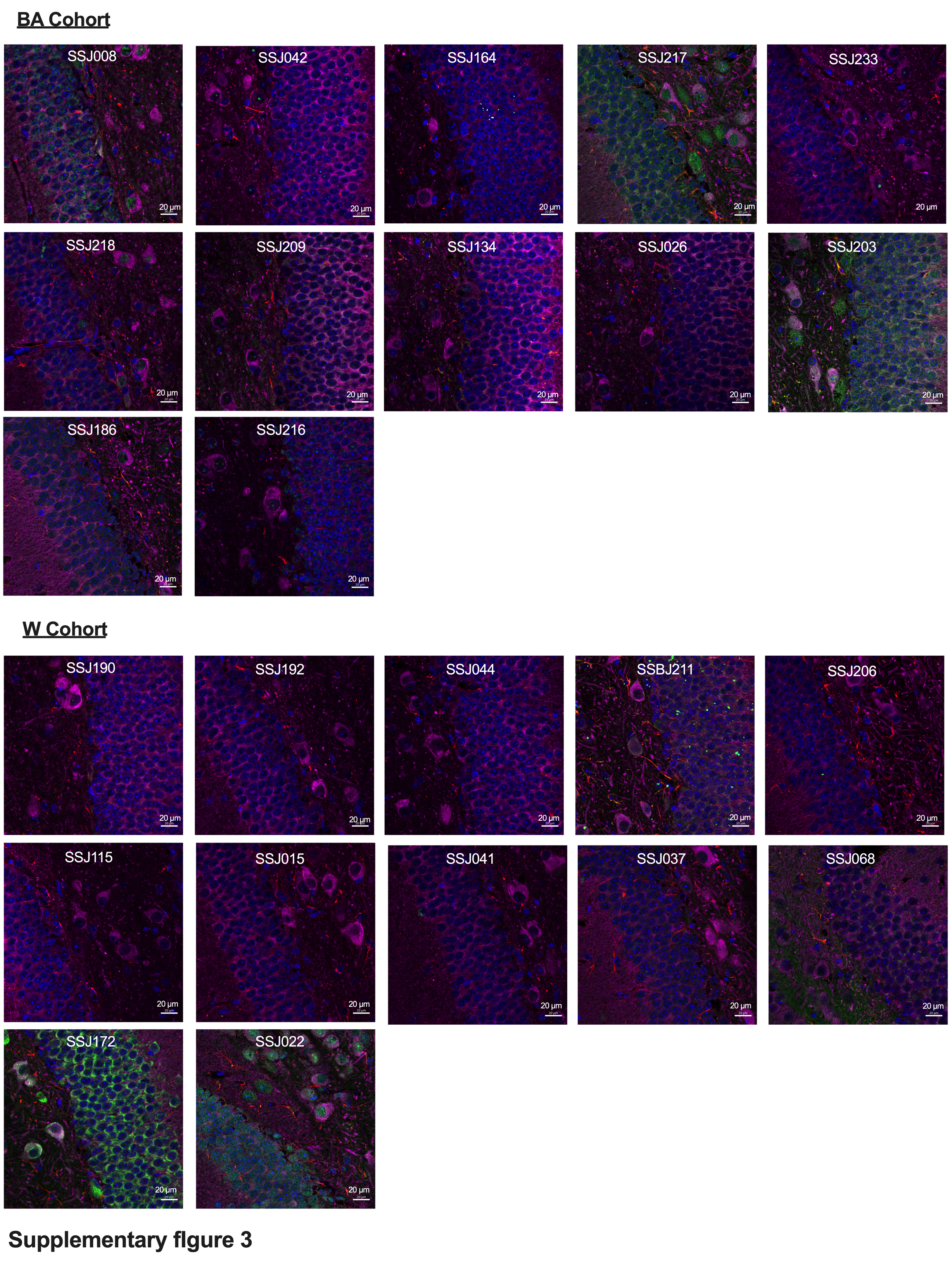
**

**Supplementary Figure 3. Representative images of purified plasma bulk IgG from study participants binding to murine brain tissue**. Plasma-purified IgG (Green, Alexafluor 488); MAP2-expressing neurons (Magenta, Alexafluor 647); GFAP+ astrocytes (Red, Alexafluor 568) cells (Blue, DAPI).

**Supplementary Table 1**.

Displayed are the results of multiple linear regression with West African Ancestry (WAFR) and either disease duration, number of Natalizumab infusions (#Natalizumab infusions), or body mass index as main effect independent variables. An interaction term for WAFR and the second independent variable was included in each model. The regression fit used Ordinary Least Squares. Two-sided p values are reported, and p values < 0.05 were considered statistically significant.
